# Reasons for missed appointments linked to a public-sector intervention targeting patients with stable chronic conditions in South Africa: results from in-depth interviews and a retrospective review of medical records

**DOI:** 10.1186/s12875-017-0655-8

**Published:** 2017-08-24

**Authors:** Bvudzai P. Magadzire, Thubelihle Mathole, Kim Ward

**Affiliations:** 1 School of Public Health, University of the Western Cape, Bellville, South Africa; 2 School of Pharmacy, University of the Western Cape, Bellville, South Africa

**Keywords:** Missed appointments, Access to medicines, Non-communicable diseases, Chronic dispensing unit, Pharmaceutical systems, Western cape, South Africa, Low-and middle-income countries

## Abstract

**Background:**

Missed appointments serve as a key indicator for adherence to therapy and as such, identifying patient reasons for this inconsistency could assist in developing programmes to improve health outcomes. In this article, we explore the reasons for missed appointments linked to a centralised dispensing system in South Africa. This system dispenses pre-packed, patient-specific medication parcels for clinically stable patients to health facilities. However, at least 8%–12% of about 300,000 parcels are not collected each month. This article aims to establish whether missed appointments for collection of medicine parcels are indicative of loss-to-follow-up and also to characterise the patient and health system factors linked to missed appointments.

**Methods:**

We applied an exploratory mixed-methods design in two overlapping research phases. This involved in-depth interviews to yield healthcare practitioners’ and patients’ experiences and medical record reviews. Data collection was conducted during the period 2014–2015. Qualitative data were analysed through a hybrid process of inductive and deductive thematic analysis which integrated data-driven and theory-driven codes. Data from medical records (*N* = 89) were analysed in MS excel using both descriptive statistics and textual descriptions.

**Results:**

Review of medical records suggests that the majority of patients (67%) who missed original appointments later presented voluntarily to obtain medicines. This could indicate a temporal effect of some barriers. The remaining 33% revealed a range of CDU implementation issues resulting from, among others, erroneous classification of patients as defaulters. Interviews with patients revealed the following reasons for missed appointments: temporary migration, forgetting appointments, work commitments and temporary switch to private care. Most healthcare practitioners confirmed these barriers to collection but perceived that some were beyond the scope of health services. In addition, healthcare practitioners also identified a lack of patient responsibility, under-utilisation of medicines and use of plural healthcare sources (e.g. traditional healers) as contributing to missed appointments.

**Conclusion:**

We suggest developing a patient care model reflecting the  local context, attention to improving CDU’s implementation processes and strengthening information systems in order to improve patient monitoring. This model presents lessons for other low-and-middle income countries with increasing need for dispensing of medicines for chronic illnesses.

**Electronic supplementary material:**

The online version of this article (doi:10.1186/s12875-017-0655-8) contains supplementary material, which is available to authorized users.

## Background

Missed clinic appointments by patients are a common phenomenon in healthcare provision globally and across different types of diseases [1–4]. The problem with missed appointments is that continuity and effectiveness of healthcare delivery is compromised, appropriate monitoring of health status lapses, and the cost of health services might increase [3]. Furthermore, some studies have shown a relationship between missed appointments and sub-optimal clinical outcomes among patients with chronic diseases [3, 5, 6]. Missed appointments serve as a key indicator for adherence to therapy and as such, identifying patient reasons for inconsistency in meeting appointments could assist in developing programmes to improve health outcomes [5].

In this article we explore the reasons for missed appointments linked to a public sector, access to medicines intervention in the Western Cape province of South Africa - the Chronic Dispensing Unit (CDU). Established in 2005, the CDU was designed to dispense medicines for clinically stable patients with HIV and/or non-communicable diseases. The CDU was born out of an increasing demand for medicines to treat chronic diseases in a context of severe healthcare practitioner (HCP) shortages, over-burdened healthcare facilities and long patient waiting times in the public sector - challenges that are widely acknowledged in other settings. The CDU was developed to overcome these challenges through the establishment of a private-sector outsourced, centralised medicines dispensary. At the time of our study, over 200 health facilities and about 300,000 patients were registered for this intervention.

In summary, the CDU process begins with the contracted service provider collecting prescriptions from health facilities. These are prescriptions of patients deemed to be stable on their therapies and therefore require minimal follow-up by clinicians. [7] The dispensing process, including prescription evaluation and interpretation and automated picking and packing ensues. These patient- and health facility- specific parcels are delivered to facilities for distribution to patients at the facility or alternative sites (e.g. community halls) [8–10].

According to legislation, a patient receives a prescription which is repeatable for up to six months and a six-month follow-up appointment with the clinician to assess therapeutic outcomes ensues. According to the CDU policy, the first issue of medicine from the eligible prescriptions is always dispensed at the health facility, and thereafter repeats (generally monthly and bi-monthly in urban and rural areas, respectively) are dispensed by the CDU. As such, beneficiaries of the CDU have monthly or bi-monthly appointments for medicines collection. In some instances, this appointment is strictly for medicines collection, while in other cases, it might include symptom screening and a brief educational session by the healthcare practitioner (HCP). Based on the clinician’s evaluation at follow-up stage, the same process described above is repeated after review. If the patient’s clinical status changes and the clinician deems regular monitoring a necessity, the patient will be referred back to mainstream care with the possibility of being enrolled with the CDU again at the clinician’s discretion.

Once patients have been enrolled, those who miss medicine-collection appointments consecutively and fail to report to the health facility within five days from date of scheduled appointment should be de-registered from the CDU and requested to consult with the HCP more regularly. De-registered patients can be offered the opportunity to enrol again at the HCP’s discretion. Designers of the CDU intervention factored in a monthly allowance of 4% missed appointments (in relation to medicines collection) to accommodate loss-to-follow-up, death or other unforeseen circumstances. This target of 4% was informed largely on the principle of economic efficiency, given that the cost of (potentially) unused medicine would be unacceptably high. While reviews of studies on missed appointments have shown much higher rates of missed appointments (up to 55% in some settings) [11], the assumption was that the CDU would target stable patients, who require minimum follow-up care [12], are adherent to clinic appointments and well controlled on treatment. In reality, at least 8 to 12% of nearly 300,000 medicine parcels are not collected every month [10]. This figure is a conservative estimate, as many facilities under-report on collection statistics. Missed appointments for medicine collection are a concern to government, not only because of associated cost and potential losses due to expired medicines but due to the additional workload that is generated for both the service provider and HCP at health facilities and the potential negative patient therapeutic outcomes [10].

The Western Cape Department of Health (WCDOH) commissioned this study to investigate the unexpected rates of missed appointments. Chen (2006) has suggested that the success of an intervention is affected by the arrangement of multiple components, including aspects of the target population, programme implementers, programme planners and community partners [11]. In this article we explore the challenge of missed appointments using a combination of routine data and perspectives and experiences of the target population (patients) and programme implementers (HCP). Specifically, we aim to establish whether missed appointments are indicative of loss-to-follow-up; and to characterise the patient and health system factors linked to missed appointments.

We focussed on type-2 diabetes and hypertension because the prevalence of these non-communicable diseases (NCDs) are highest in the CDU population and because of their global prominence [12–14].

## Methods

### Study design

We applied an exploratory, cross-sectional mixed-methods design in two overlapping research phases. This involved in-depth interviews to yield HCP and patients’ experiences, perceptions and feelings [15]. In addition, we conducted medical record reviews (MRR) in order to establish whether missed appointments were temporary or indicative of loss-to-follow-up. Data collection was conducted during the period 2014–2015 after getting ethical clearance from the Senate Research Committee at the University of the Western Cape, South Africa and permission to do research from the relevant structures within WCDOH.

### Characteristics of the patient population

The majority of the South African population (more than 75%) is dependent on the public sector for, inter alia, supply of medicines [16] and CDU patients are part of this constituent. This research was conducted in relatively indigent areas (Gugulethu, Mfuleni, Mitchells Plain and Khayelitsha).

### Facility and participant selection

In early 2015, the CDU supported approximately 77% of the total PHC facilities in the province, which included 44 urban facilities administered by the WCDoH. In most cases, diagnosis of NCDs and stabilisation on chronic medicine occurs at this level [17]. For this study, we focused on urban community health centres (CHCs) where the CDU roll-out was initiated and is therefore better established. Facilities were categorized according to monthly medicine parcels received from the CDU: less than 4000 (small); 4000–10,000 (medium) and more than 10,000 (large). These were then stratified according to the history of reported missed appointments. For variability of experiences, we selected one small-, two medium- and one large site(s) – two with a documented history of missed appointments and two without. During the time of the research, there were 210,296 active CDU beneficiaries, of which more than 80% were over 40 years of age and about 66% were female [10]. The four selected facilities represented approximately 10% of the total active CDU population.

Participants for in-depth interviews were sampled purposively as follows:

- **Patients:** type-2 diabetes and/or hypertension patients who had missed their previous CDU appointment (*N* = 23). All patients had voluntarily presented at the facility and were identified by HCP during the triaging process.
- **HCP:** provincial mid-and senior-level managers involved in the care continuum (*N* = 9); frontline HCP - physicians, nurses, pharmacists and health promoters (*N* = 22).
- **CDU service provider:** (*N* = 4).

Medical record reviews were conducted at two out of the four facilities with a well-functioning electronic pharmaceutical system to allow for follow-up [18]. This approach was employed in order to establish whether patients were lost-to-follow up. This included establishing the time taken by patients from the time of scheduled (missed) appointment to the time of next visit or establishing whether the patient was lost-to-follow-up. In addition, demographic characteristics of the patients (age, gender) were obtained.

### Data collection processes and tools

***Phase 1a*** **:** For the MRR, we selected a sample of 112 CDU patients who had missed their appointments on two randomly selected days in May and June 2014. We followed-up two months after the date of missed appointment. A template was developed in Microsoft Excel to capture patients’ identifiers. These details were used for MRR that was done by a pharmacist at two out of the four health facilities. During this exercise, the pharmacist captured patient age, diagnosis, last date of presentation prior to or after the missed appointment and outcomes at last assessment.

***Phase 1b*** **:** We targeted patients who had missed their previous appointment using type-2 diabetes and hypertension as the primary tracer conditions. The tool used in this phase was guided by selected constructs adapted from the Explanatory Model Interview Catalogue (EMIC). This is a cultural epidemiology framework which aims to capture descriptive accounts of local interpretations of illness, its meaning and associated illness behaviour, and emphasises the need for a holistic assessment of the patient, including the role of culture in long-term illnesses [19].

In addition, we captured reasons for missing medicine collection appointments. The EMIC framework allowed us to obtain reasons for missed appointments and to contextualise patient behaviour beyond the immediate reasons for missing appointments. As such, we profiled patients’ illness experiences, focussing on path to diagnosis, access to services and/or information, self-treatment, treatment experience and social support, as any one or all of these factors might influence behaviour. Tools were tested in a pilot study and subsequently refined (see Additional file 1).

***Phase 2:*** Semi-structured interview guides were developed for interviews with provincial managers and frontline HCP and focussed on patient-related and health system factors contributing to missed appointments (see Additional file 2).

Interviews were conducted face-to-face, except for one respondent who relocated to another province and completed a telephonic interview. Interviews were conducted at participant’s work settings or at CHC in the case of patients and frontline HCP. Interviews with HCP were conducted by the first author, who is a qualitative researcher with a background in public health. Once no new information was generated from the interviews (saturation), no further interviews were conducted. All participants were taken through the informed consent procedure prior to interviewing, including a request to record the interview if the participant was willing. Three participants refused to be recorded and notes were taken for those interviews. Participants were also informed of their right to withdraw at any time without any consequences.

Medical record reviews (MMR) were conducted by a pharmacist and patient interviews were conducted by a trained fieldworker proficient in local vernaculars.

### Data analysis

Data from MRR were analysed in MS Excel using both descriptive statistics and textual descriptions. Twenty-two folders were excluded from the final analysis as a result of incomplete data (*N* = 20) and capturing error (*N* = 2). This resulted in a final sample of 89.

For the qualitative interviews, data analysis was, as is common in qualitative research, a continuous process [15]. This meant that issues of relevance emerging from an interview were included in subsequent interviews.

The audio-taped interviews were transcribed verbatim and patient interviews were translated into English. Thematic analysis [20] was used to analyse qualitative data. This process involved a hybrid process of inductive and deductive thematic analysis [21] which integrated data-driven with theory-driven codes based on the domains of the EMIC framework.

The data was then coded, compared and contrasted, and recurring elements were matched to generate categories which were further collapsed to form themes that are presented in this article. Atlas.TI version 7 was used to organise the data. The transcripts were independently read by the first author, and emerging themes were discussed with all authors. Further analysis (drawing relevant meanings, and searching for relationships among and within data) was then conducted. Finally, the results were discussed with selected participants through feedback sessions with HCP and patients as a way of conducting participant validation [22].

## Results

### Status of a sub-sample of patients who missed appointments

**Fig. 1:**
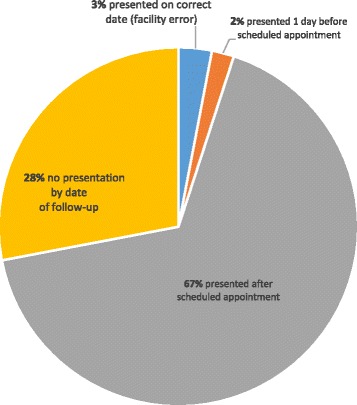
Presentation status of patients who missed appointment dates (*N* = 89)

The majority of patients who missed appointments were female (66%), with a median age of 56, suffering from mainly diabetes and/or hypertension (Table 1). This aligns to well to the characteristics of the general CDU population which was shown in a previous analysis to be predominantly female (66%) and over 40 years of age (+80%) [10]. During the MRR, we identified four categories of patients, i.e. (i) those who presented after their scheduled appointment; (ii) those who had not presented by the time of the study; (iii) those who presented on the correct date but were (erroneously) recorded as defaulters and (iv) those who presented earlier than the scheduled appointment. Figure 1 represents the different proportions for each category. The majority of patients who missed appointments (67%) reported to the facility after the scheduled date (Fig. 1) - times ranged from 1 to 84 days with a median of 28. However, 28% had not reported to the facility by the time of the study and times ranged from 10 to 149 days with a median of 56. A small percentage (3%) presented on the correct date while even fewer (2%) presented a day early.

**Table 1:** Characteristics of patients sampled for medical record reviews

Gender	Female (66)
	Male (23)
Age (years)	Range: (23–86)
	Median age: 56
Chronic condition (not exclusive, some patients had multiple morbidities)	Diabetes (34%)
	Hypertension (73%)
	Diabetes and hypertension co-morbidity (34%)
	Others, e.g. chronic obstructive pulmonary disease (56%)

Results pointed to the need to distinguish between true defaulters and others who were misclassified as a result of inefficient facility processes. The latter was evidenced by patients who attended the CHC on the correct date or within the permitted time-frame (≤3 days before or ≤5 days after) but obtained medicines from the traditional (internal) pharmacy system instead of their CDU parcel. Also, the median number of days between a missed appointment and the next or most recent date of presentation (28 and 56 respectively), suggests that some patients who kept appointments may have received medicines through the traditional pharmacy system, given that patients operate on a 28 and 56–day medicine collection appointment cycle.

Further assessment of medical records for patients who had not reported to the facility by date of follow-up (*N* = 24), revealed other possible health system inefficiencies which contributed to missed appointments. For example, two patients had been transferred to other facilities but their parcels had not been cancelled at the CDU. Healthcare practitioners suggested that either the cancellation process had not been carried out or was delayed, hence the parcels were still delivered to the facility. Another example was that of a patient who was newly-diagnosed and enrolled onto the programme - contrary to the CDU policy of enrolling only patients who have been stabilised on therapy. Eight patients collected medicines through the internal pharmacy system and followed a different appointment system. One of the reasons offered by HCP was that these patients could have reported for acute conditions during their treatment trajectory and when they received treatment for acute conditions, they were also given chronic medication through the pharmacy, hence another treatment cycle began which was not in sync with their CDU appointments. Healthcare practitioners did this to  to reduce the frequency of patient visits to the facility and to minimize the  associated costs of transport.

With the remaining 13 (out of 24) patients, it was not clear why they had not returned to the facility. However, 11 of them were not clinically stable based on local diabetes and hypertension guidelines for at least one condition at last assessment, therefore HCP suspected that they could have been hospitalised or died. Existing information systems were not synchronised to flag patients hospitalized elsewhere or even when collection had been done through the traditional pharmacy system in the same facility. Attempts to contact patients who had not presented by the date of follow-up were unsuccessful because of missing or wrong personal information.

In summary, from an analysis of this small population, the majority of patients (72%) were not lost-to-follow-up; they were either misclassified as a defaulter or encountered individual or health system related barriers, all of which need to be better understood.

### Reasons for missed appointments – Insights from in-depth interviews with patients and healthcare practitioners

The majority of patients in this sample were female (91%), with a median age of 54, and most had multi-morbidities (Table 2).

**Table 2:** Demographic characteristics of patients interviewed *N* = 23

Gender	Female (21)
	Male (2)
Marital Status	Widowed (8)
	Married/Co-habiting (8)
	Divorced/Separated (3)
	Never married (4)
Highest level of education obtained	Secondary (23)
Employment status	Not working (11)
	Informal employment (12)
Age (years)	Range: 35–83
	Median: 54
Number of self-reported chronic conditions	Range: 1–4
	Median: 2
Self-reported duration on treatment	Less than five years (6)
	Six-ten years (7)
	More than ten years (10)

The immediate reasons for missed appointments as expressed by patients were: mobility and temporary migration, forgetting or mixing up of appointments, work commitments and switch to private medical care.

Of these, the most common reason for missed appointments was mobility and temporary migration. Given that the Western Cape is an economic base for people from other South African provinces, it is common for people to travel back and forth from their provinces of origin for planned holidays (especially during the festive season), religious activities and employment seeking as was reported by study participants or unplanned events such as funerals. At least seven patients indicated that their appointment was at a time when they were travelling to or already in another province.

The second most common reason for missed appointments as expressed by patients was forgetting or mixing-up appointment dates partly because of poorly written appointment cards. A Short Message Service (SMS) appointment reminder system had been designed as a strategy to mitigate this challenge. However, we did not establish whether patients who were subscribed to the SMS reminder system adhered to their appointments better than those who were not. What was clear was that this service benefited only a small number of patients. The service provider estimated that of between 13,000 and 14,000 parcels that were dispatched to facilities daily, only about 12% were linked to contact numbers and of that proportion, 15% of the messages were undeliverable. Maintaining an up-to-date database of contacts at the facility level proved challenging. Furthermore, some facilities had voluntarily unsubscribed from the service because it created confusion among patients when the messages where misinterpreted by patients.

The third factor causing missed appointments was linked to work commitments. Unskilled workers such as domestic workers who reside with their employers faced the greatest challenge. Our results suggest that their jobs might have limited flexibility hence they rely on others, such as family members, to collect their medication. Similarly, reliance on family members or even other community members was a coping strategy for the elderly with debilitating illness, another cause for missed appointments. As one elderly female said:

*“Today, I had to come myself but I used to send a young man from the neighbourhood…now he died so I have no one to take my medication.” [P13, 83- year old].*

Despite making prior arrangements, this strategy can fail due to other barriers at the facility level. Speaking on this issue, one patient said:

*“…once, I sent my grandchild to pick up the medicine for me and they did not give her. They said they wanted my original ID.” (P10, 71- year old).*

The last reason for missed appointments, i.e. temporary migration to private medical care, was mentioned by an elderly patient (77 years old). The patient’s daughter had enrolled her onto a private medical scheme because it was perceived that she could obtain better quality of care in the private sector. However, she was still registered on the public system and returned when private funds were depleted. Although an uncommon reason for missed appointments in this study, migration to the private sector flagged some dissatisfaction with public health services, an important contributor to missed appointments. The patient in question complained of long waiting times at her local CHC. Other patients also cited long waiting times at the pharmacy:

*“The pharmacy should try to speed up things there or do something also because that is where I wait long time.” (P6, 43-year old).*

In contrast, other patients accepted long waiting times as the status quo:

*“It can’t be a concern because that’s how our clinics operate.” (P1, 61-year old).*

*“It’s something that is so common in waiting and crowding is something that I have accepted and it’s no concern.” (P22, 59-year old).*

Healthcare practitioners gave additional reasons for missed appointments. They concurred that long waiting times for medicines collection could be a contributor to missed appointments. In two of the sampled facilities, HCP mentioned that the patient load was still significantly high, with waiting times of five hours or more at the pharmacy alone despite some efforts to decongest CHCs by introducing alternative medicines distribution strategies.

In addition to the reasons for missed appointments given by patients above, some HCP felt addressing reasons for missed appointments was complicated by patients who were perceived to give ‘socially desirable’ responses when questioned about reasons for missing appointments. For example, travelling to another province for a funeral would possibly garner more sympathy than admitting to having forgotten. They also expressed concern about patient attitudes, such as perceived lack of patient responsibility and under-utilisation of medicines (leading to over-supply) which they felt also contributed to missed appointments. Triangulation of this view with observations and patients’ treatment experiences which were captured through the EMIC constructs, did show possible underutilization of medicines. An incident was recorded in the field by the first author, where family members of a deceased patient returned a large bag of unused medicines. This was confirmed by HCP to be a regular occurrence because some patients did not take the medicines as prescribed. Lastly, the use of plural healthcare sources emerged as an important aspect of some patients’ treatment experiences. Specifically, self treatment with alternative medication sourced through informal providers (herbalists and traditional healers) was reported. Some of the remedies that were obtained from informal providers were described by patients as “*cleansing the body and purifying the blood*” or “*calms the hypertension*”. We inferred that the use of plural healthcare sources could result in missed appointments because patients could under-prioritise their clinic appointments.

### Looking ahead

Overall, the range of access barriers was not adequately taken into account during the design phase of the CDU intervention, hence there were limited strategies to address them. Our results suggest that missed appointments emanate from a combination of individual and health system barriers. Also, some cases recorded as missed appointments emanate from inefficiencies in implementation of the intervention, an issue requiring the attention of all stakeholders involved in the CDU process. The individual barriers, however, while acknowledged by HCP, were perceived to be beyond the scope of care provided by health services; pointing to the link between health and welfare and the necessity of inter-sectoral strategies as illustrated in the quotes below:

*“The clinician can only do so much in interventions – some of what is required are socio-economic interventions that deal with societal factors and lifestyle changes.” [Physician].*

A health manager also said:

*“Health can do so much; inter-sectoral collaboration is required because poverty plays a role, your circumstances, economic factors and all of that.”*

*[Senior Manager, WCDoH].*

## Discussion

Driven by the need to address the challenge of missed appointments by beneficiaries of the CDU in South Africa, this study begins to question some of the initial assumptions made about this intervention. Programme planners envisaged that beneficiaries would be adherent to their appointments (with an allowance of 4% defaulter rate monthly), adherent to medication and ultimately exhibit stable clinical outcomes. In a previous study, we established that rates of missed appointments were higher than 4% [10] and in this study, there was some evidence of non-adherence to medication (although this was not a central focus of the study) and some patients exhibited unstable clinical outcomes. Also, some cases recorded as missed appointments are not truly so, rather they emanate from inefficiencies in implementation of the intervention, which is a separate issue.

### Role of context factors in the chronic illness experience

We identified individual patient factors tied to contextual realities which impacted on medicines collection and potentially on clinical outcomes. These included forgetting appointments and work commitments for people in the informal sector and other factors that are well described in literature [23, 24]. Through the use of the EMIC framework [19], we were able to also identify potential threats to patients’ keeping appointments such as those related to self-treatment.

In our view, although some issues have been acknowledged in literature, their incorporation into frameworks or strategies designed to improve access to medicines finds little space in the NCD literature and were not adequately considered during the design of this intervention. This is evidence that chronic disease management extends beyond mechanistic implementation of purely technical interventions such as the CDU [25], and that failure to address social factors impacting on patients leads to failure to effectively serve the population. From our study, HCP’s mistrust of patients’ reasons for missing appointments could also influence how they react to patients. In line with our findings, a recent study in the United States of America found a discrepancy between the identifiable social factors and health providers’ ability to address them [26].

Notably, despite prevailing challenges, most patients – and all interviewed in this study - who missed appointments returned to the CHC without follow-up. Similarly, over 60% of patients who were included in the folder review also reported to the CHC, albeit late. This could be an indication of some degree of patients’ willingness to cooperate with the policies of the intervention. However, it presents challenges given that patients can go for an indefinite period without medication, which can affect their health status. Other studies have reported that an opportunity for patients to cancel/postpone appointments can lessen missed appointments [27] and inform the development of strategies to better address the issues faced by patients. In the case of the CDU, a two-way communication system is lacking to allow patients to communicate with health services if they cannot make it for an appointment.

### Contribution of CDU implementation-related factors

Through our study, we were able to identify problematic process-related issues contributing to missed appointments. It was clear that there were some patients whose parcels were returned to the CDU yet they attended the CHC and received medicines via the parallel dispensing system, at times on the correct date or within the permitted time frame. Also, incidents showing that transferred patients and/or those who no longer required medication were not easily identifiable pointed to the need for integrated information systems. In addition, issues such as enrolment of newly diagnosed patients and those not well controlled on therapy who were kept on the programme, called into question the overall patient management strategy. More specifically, the 14% classified as poorly controlled on therapy at last assessment were at risk of being lost-to-follow-up yet there was no information about their status. Such patients’ circumstances may have predisposed them to needing more care than the health system provided or they required linkage to non-health services. These findings seemed to support the view that unexpected results could be due to an intervention theory that has not been carried out well; or the problem could be with the theory itself [28]. This study presented evidence on underlying implementation problems which require further study in order to unravel the interplay of multiple components of the intervention [11].

### Strengths and limitations of this study

This study is part of a service improvement process and the findings were disseminated to relevant stakeholders for immediate consideration. We highlight an oversight on the role of social factors and also make a case for an in-depth process evaluation of CDU implementation. The study also has some limitations. Our results should be interpreted in context as they might not be reflective of the range of barriers faced by CDU patients because we captured reasons for missed appointments linked to a specific event. Also, the majority of respondents in the patient population were female. This was not surprising given that the majority of beneficiaries are female. However, their experience might not be reflective of their male counterparts.

### Implications for policy and practice

Upon reflection, an imperative issue was whether the 4% rate of missed appointments assumed by the service was appropriate for this context. This was not easy to answer given that some missed appointments were a result of health system inefficiencies not actual missed appointments. However, the MRR still showed at least 28% who were lost-to-follow-up. Furthermore, for this type of intervention, late presentation by patients and misclassification has implications on implementation costs.

There are some barriers that continue to perpertuate missed appointments. Currently, the SMS reminder service exists to reduce missed appointments caused by forgetting of appointments. Similar interventions have  been reported to improve attendance [29], however, it was not clear if this was the case with the CDU. That said, SMS reminders would only solve part of the problem.

This article concludes by highlighting three priorities that could address the problem of missed appointments and for the broader management of NCDs in our context. These issues are also of relevance to other stakeholders seeking to strengthen health systems by provision of differentiated care for patient groups in other contexts.

First, the assumptions made about patients and the health system are not aligned with the reality. Socio-economic factors affect health facility attendance in the long run and invariably undermine health status. An appropriate method for establishing these barriers must be found in order to improve management of at-risk patients and to inform the development of contextual approaches to addressing public health problems [30]. This could be achieved by undertaking population-based studies to inform the development of an appropriate, patient-centred package of care. For example, while labour migration is common throughout South Africa and the southern African region, the Western Cape is unique in that the majority of its migrants come from and regularly visit the same part of South Africa (the Eastern Cape). This provides an opportunity to implement patient support strategies specific to this migration route to ensure that patients on chronic medication can be assisted to timeously collect their medication. The NCD care package currently fails to normalise travel as an expected part of many patients’ lives.

Second, routine cohort monitoring of patients is essential. Currently clinical outcomes are recorded using a paper-based system in the public sector. Consequently, there is no opportunity to monitor the cohort in order to identify patients who might be defaulting or lost-to-follow-up. Such information can inform health managers about patient status. There is a huge potential to improve NCD care through eHealth [14]. Given the availability of affordable mobile electronic options, the incorporation of electronic medical record technology appears to hold promise for the rapid implementation for monitoring of NCD cohorts [31]. A combination of an efficient electronic system and support for systematic collection of routine NCD data is likely to yield positive results.

Third, it is necessary to revisit the patient care model as a whole to ensure that it is responsive to the needs and realities of the patients. If care for stable and at-risk patients is to be differentiated, then appropriateness of the care package and quality of care become critical elements of service delivery. A number of issues raised in this study are relevant in the development of an appropriate package that responds to patient and health system needs. To date, we see pockets of interventions [32, 33] but no comprehensive approach. While these are useful, a single factor approach will likely have limited effectiveness. Given the shared barriers and challenges faced by health programmes, some of the approaches developed for HIV programmes have the potential to contribute to models needed to address NCDs in resource-limited settings [34]. We acknowledge that HIV programmes have been fairly well resourced compared to NCDs; however, there is opportunity to adapt interventions to available resources.

## Conclusion

The challenges highlighted in this article should not underplay the contribution of the CDU as a useful intervention to address patient and health system barriers to accessing medicines. We suggest developing a model of care reflecting local context, attention to improving CDU’s implementation processes and strengthening information systems inorder to improve patient monitoring. The CDU presents lessons for other low-and-middle income countries with increasing need for dispensing of medicines for chronic illnesses.

## Additional files

Additional file 1: Patient SS Tool_30_07_2014. Patient data collection tool. (PDF 533 kb)

Additional file 2: Interview Schedule_KIs_GENERIC. Semi-structured interview guide for key informants. (PDF 137 kb)

## Abbreviations

CDU: Chronic Dispensing Unit
HCP: Health Care Practitioners
NCDs: Non-Communicable Diseases
PHC: Primary Healthcare
WCDoH: Western Cape Department of Health
WHO: World Health Organisation
