# Supplementary material for: Reasons for missed appointments linked to a public-sector intervention targeting patients with stable chronic conditions in South Africa: results from in-depth interviews and a retrospective review of medical records
Source: BMC Fam Pract. 2017 Aug 24;18:82. doi: 10.1186/s12875-017-0655-8 (PMC5571491; doi:10.1186/s12875-017-0655-8)
Supplement: Supplementary file 1 — Patient SS Tool_30_07_2014. Patient data collection tool. (PDF 533 kb) [file 12875_2017_655_MOESM1_ESM.pdf]

Patient-User Survey: GENERIC

SITE

Date of interview | Day | Month | Year |

**Respondent ID**		**Clinic or group name**	
**Condition**		**Location**	
**Interview Start Time**		**Urban/Peri-urban/Rural**	*1 Urban* / *2 Rural*

"To begin, we would like to ask you some general questions about yourself."

RESPONDENT CHARACTERISTICS

1. Sex | *1* F | *2* M |

2. Age | |

3. Head of household? | *1* Yes | *0* No |

4. Number in household | |

5. **Marital status** *Tick one only*

1 Never married	*2 Married*	*3 Co-habitating*	*4 Separated*	*5 Divorced*	*6 Widowed*

6. **Place of residence:** ____________________________________

____

(if residence is different from clinic location, probe for reasons)

1 Urban	*2 Peri-urban*	*3 Rural*

7. **Employment status** *Tick one only*

1 Not working	*2 Retired*	*3 Student*	*4 Seasonal Work*	*5 Part-time Work*	*6 Full-time Work*

8. **Occupation** *Tick one only*

Specify ___________________________

1 Unskilled labour	*2 Vocational labour*	*3 Small business owner or manager*	*4 Professional*
			5 Other

9. **Personal income per month** | |

9.1 **"Is this income regular for most months?"** | *1 Yes* | *0 No* |

Tick one only

10. **Household income per month** | |

10.1 **"Are you/ or is someone in your household receiving a social grant?"**

1 Yes<br>*Child*<br>*Disability*<br>*Older persons*<br>*Veteran*	*0 No*

"What is your highest level of education completed?" *Tick one only*

1 No Schooling	*2 Primary school*	*3 Secondary school*	*4 Vocational school*	*5 University*	*6 Post-graduate*

11. **Religion**

1 Protestant	*2 Catholic*	*3 Muslim*	*4 Hindu*	*5 None*	*6 Other (specify):*

ILLNESS EXPERIENCE and EXPLANATORY MODELS

"Now, we would like to ask you some general questions about your health."

12. **"What** *(Tick all that apply)* **are the symptoms/problems for which you first came to this clinic for help?"**

Narrative: ____

____

____

Tick spontaneous in 12.1 for any that apply

12.1 "**Have you experienced any of the following symptoms**?" *Tick probe for all that apply*
Clarify with respondent to ensure reference to presenting condition, not side effects of treatment

**Patterns of Distress** *(conditions that it applies to)*	**Spon**	**Probe**
1 Headaches		
2 Thirst		
3 Regular urination		
4 Bodily pain		
5 Weight change (loss or gain)		
6 Weakness		
7 Poor appetite		

**Patterns of Distress**	**Spon**	**Probe**
8 Nausea & vomiting		
9 Sleep problems		
10 Emotional distress		
11 Skin lesions or discoloration		
12 Chest pains & difficulties breathing		
13 Others(write in):		

12.2 **"Of those symptoms you mentioned, which one is the most troubling for you?**
Code single most troubling symptom from above numbered list

13. **"What name do you give the condition for which you are seeking treatment?"**
Write name in field and refer to this term in all following questions

Narrative____

____

____

14. **"How long ago were you diagnosed?**
Here you can also insert month & year e.g. if client says Jan 2014

Years	*months*	*days*

15. **"People may have various ways of explaining what causes their illness, which may be different from what their doctors or family think.**

What do you think caused you to have [condition]?"

Narrative: ____

____

____

Tick 'Spon' in grid below for all spontaneously reported Perceived Causes reported

15.1 "**Have any of the following issues caused [condition]?"**
Ask respondents about categories not already mentioned spontaneously. Code "probe" for all that apply

**Perceived cause** *(conditions that it applies to)*	**Spon**	**Probe**
1 Personal habits - smoking, drinking, drug-taking (HIV, Diabetes, HPT, Depression)		
2 Something in body has gone bad (Diabetes, HPT, Depression)		
3 Something I ate (HIV, Diabetes, HPT, Depression)		
4 Spiritual or magical forces (HIV, Diabetes, HPT, Depression)		

**Perceived cause**	**Spon**	**Probe**
5 Family history (HIV, Diabetes, HPT, Depression)		
6 Other (write in):		
7 Cannot say		

15.2 **"Of the causes you mentioned, which one is the most important cause of your [condition]?"**

Code single most important Perceived Cause from above numbered list ☐

Narrative: ____

____

____

16. **"Are there any other health problems that you are currently experiencing?"**

List all conditions beside number

1	*4*
2	*5*
3	*6*

16.1 **"Are any of the conditions just described related in any way to [state previous condition]?"**

3 Yes	*2 Possibly*	*1 Uncertain*	*0 No*

Tick one only

Narrative: ____

____

____

HELP-SEEKING

"Next, we would like to ask you some general questions about how you have sought help for the condition."

17. **"When you first recognized [condition] as a problem, did you do anything to help yourself at home? If so, what did you do?"**

Narrative: ____

____

____

Tick 'spontaneous below for all that apply

17.1 "Did you do any of the following…?"

Probe for categories not previously mentioned. Tick all that apply

**Help-Seeking (home)**	**Spon**	**Probe**
1 Self-treatment with home remedies		
2 Self-treatment with medicines that you already had at home		
3 Changed daily habits		
4 Changed eating habits		

**Help-Seeking (home)**	**Spon**	**Probe**
12 Sought family advice/assistance		
13 Sought friend's advice/assitance		
7 Prayer		
8 Other (specify):		

18. **"Many people seek help from various sources, not just doctors. What other kinds of help outside the home have you used?"** *Tick all spontaneous responses and then probe for categories not mentioned*

**Help-Seeking (outside)**	**Spon**	**Probe**
1 Herbalist		
2 Spiritual healer		
3 Community-based health worker		
4 Public primary healthcare facility (e.g. Clinic)		
5 ~~Rural hospital~~		
6 District level hospital		
7 Private clinic or GP/doctor		

**Help-Seeking (outside)**	**Spon**	**Probe**
8 Nursing home		
9 Church, mosque, temple		
10 Pharmacy		
11 Health support group		
12 Other(specify):		

18.1 **"Of those sources of help you mentioned, to which did you go first?"**
Code the first source of help sought from above numbered list ☐

Narrative about first source and any subsequent sources mentioned

____

____

____

19. **"How long after your first became aware of the initial symptoms of [condition] did you first seek help from this source?"** *Refer tor the source in question 18*

years	*months*	*days*	immediately

19.1**"Did you receive the diagnosis for [condition] from this source?"**
Note that diagnostic term might not be the same as from the clinic

1 Yes	*0 No*

19.2 If no to 19.1,
"From which source of help you mentioned did you receive your current diagnosis? Or was it <u>this</u> clinic?" *Code number from grid in Q18 or code '99' for this clinic* ☐

"Now we would like to ask you about seeking help at This Clinic."

20. **"How long ago did you start coming to this clinic (or group) for your [condition]?"** *Refer to the clinic or group from which patient was recruited*

years	*months*	*days*

21. **How far away do you live from this clinic (group)?**

Less than 5km	*5-10km*
More than 10km	*Cannot say*

22. **"How long does it usually take you to get to this clinic (or group)?"** *Specify approximate time in hours and minutes, or "cannot say"*

Less than 30 min	*30min-1 hour*
More than 1 hour	*Cannot say*

23. **"How do you travel to this clinic (or group)?"**

3 Other	*2 Drive*	*1 Bus/Taxi/Train*	*0 Walk*

24. **"Are there any difficulties getting to this clinic (or group)?"**

3 Always	*2 Frequently*	*1 Occasionally*	*0 Never*

Tick one only

Narrative: ____

____

____

25. **"Do you have any concerns about the way services are provided at this clinic (or group)? If so, how serious?"**

2 Serious concerns	*1 Minor concerns*	*0 No concerns*

Tick one only

Narrative ____

____

____

Tick spontaneous below and probe for other issues not mentioned spontaneously, ticking all that apply

25.1 **"Have you encountered any of the following problems at this clinic (or group)?"**

**Concerns about health service**	**Spon**	**Probe**
1 Long waiting time		
2 Not enough privacy		
3 Doctor or pharmacist is not available		
4 Clinic is not well kempt		

**Concerns about health service**	**Spon**	**Probe**
5 Crowding		
6 Inconvenient hours of opening		
7 Other (specify):		

25.2 **"Of the things you mentioned, which one is the biggest concern for you?** *(Code from above list)* ☐

Narrative ____

____

AWARENESS and USE OF MEDICINES

"We would now like to ask you about medicines."

26. **"How long have you been taking your treatment?"**
Note: Client may say I started in 2012 then note the year.

years	*months*	*days*

Narrative____

____

27. **"From which of the following did you first hear about [these medicines]?"**

1 This clinic or group	8 Spiritual healer
2 Another clinic or group	9 TV, radio or Internet
3 Pharmacist	10 Newspaper or magazine
4 Community health worker	11 Mobile phone message
5 Spouse/Family member	12 Other (specify):
6 Friend / colleague	13 Can't say
7 Herbalist	

Code the first source of information from above numbered list [ ]

28. **"From which of the following sources, if any, have you received information about using these medicines?"**

Tick all that apply

1 Doctor		8 Spiritual healer	
2 Clinic Nurse		9 TV, radio or Internet	
3 Community health worker		10 Newspaper or magazine	
4 Pharmacist		11 Mobile phone message	
5 Spouse/Family member		12 Other (specify):	
6 Friend / colleague			
7 Herbalist			

Narrative: ____

____

____

28. **"Do the medicines usually work well for you?"**

3 Don't know	2 Always	1 Sometimes	0 Never

Tick one only

Narrative____

____

____

29. **"How do you feel about the particular features of [the medicines]: Its size, form, and colour?"**

Tick one only for each

	Like	Dislike	No opinion	Give reasons below:
29.1 Size	2	1	0	
29.2 Form *(pill vs. capsule vs. injection)*	2	1	0	
29.3 Colour	2	1	0	

Elaborate reasons: ____

____

____

30. **"Has it been difficult to take your medicines as prescribed? If so, how difficult?"**

2 Very Difficult	1 Somewhat difficult	0 Not at all difficult

Tick one only

Narrative ____

____

31. **"Have you ever had any of the following concerns about your medicines?"**

Tick all that apply

**Medicine-specific ideas**	
1 Not certain about intended effects	
2 Harmful side-effects	
3 May be fake medicine	
4 Not good quality	
5 Too many pills to take	
6 Body cannot tolerate it	

**Medicine-specific ideas**	
7 Difficult to remembert to take it	
8 Have to change eating habits	
9 Have to change other daily habits	
10 Worry about what other people will say who know I am taking this medicine	
11 Other (specify):	

31.1 **"Please explain how you have dealt with these concerns"**

____

____

____

PREFERENCE FOR TREATMENT

32. **"Have you used your medicines for any other reason apart from treating [condition]?"**

3 Yes	*2 Possibly*	*1 Uncertain*	*0 No*

Tick one only

32.1 *Write reason(s) here:* ________________

33. **"Are you aware of any alternative treatments for [condition] apart from the medicines that you are getting]?"**

3 Yes	*2 Possibly*	*1 Uncertain*	*0 No*

Tick one only

33.1 If yes,
"What are they?" *List in grid below beside number*

33.2 **"Have you used them?"** *Tick yes or no for all named under Use*

**Alternative treatment**	**Use** 1=Yes	0=No
1		
2		
3		

**Alternative treatment**	**Use** 1=Yes	0=No
4		
5		
6		

33.3 **"Would you prefer any of these over your medicines? If so, which is the most preferred?"**

Enter code number from above or '0' if no preferred alternatives are named ☐

Narrative: ____

____

33.4 **"What changes have you observed since you started taking treatment for [condition]?**
Narrative: ____

____

ACCESS to TREATMENT

"Next, we want to know about how you get medicines, including costs and other expenses you might have."

34. **"First, from where do you normally obtain your medicines?"**

Narrative: ____

____

____

Tick normally below for all that apply

34.1 **"Do you occasionally get your medicines from any of the following sources?"**
Probe for sources mentioned and tick occasionally for all that apply

**Points of medicine access**	**Normally**	**Occasionall y**
1 This Clinic or Group		
2 Pharmacy		
4 Another clinic		
5 Public hospital e.g. Groote Schuur		
6 Private clinic/GP		

**Points of medicine access**	**Noramally**	**Occasionally**
7 Support group/Club in the community		
8 Herbalist		
9 Spiritual healer		
10 Family member		
11 Other (specify):		

Narrative: ____

____

35. **"Is it usually difficult to obtain medicines when you need them?"** *i.e. coming to the clinic for your appointment*

2 Usually difficult	*1 Sometimes difficult*	*0 Never difficult*

Tick one only

Narrative: ____

____

35.1 **"Do you occasionally experience the following challenges with regards to collecting your medicines?"**
Probe for sources mentioned and tick occasionally for all that apply

**Points of medicine access**	**Normally**	**Occasionally**
1 Too sick to come to the clinic		
2 Forget my appointment		
3 Travel to visit family in another place e.g. E Cape		
4 No transport money to come to the clinic		
5 I feel well and do not need the treatment		
6 My job cannot allow me to come for my appointment		

**Points of medicine access**	**Noramally**	**Occasionally**
6 Too busy with other things		
7 No one to send for collection		
8 I have enough medicines at home and do not need to collect (*probe for excess stock*)		
9 Weather makes it difficult to come to the clinic		
10 Frequency of appointments too much		
11 Other (specify):		

35.2 How do you deal with these challenges?

____

____

____

35.3 "Have you missed an appointment or presented late to collect your medicines in the past 3 months?"

2 Cannot remember	*1 No*	*0 Yes*

35.4 If yes what was/were the reason (s)?

____

____

35.5 What did you do?

____

____

36 "Have you visited the clinic for anything else other than to collect your [condition] medicines in the past 3 months?"

2 Cannot remember	*1 No*	*0 Yes*

36.1 If yes what was/were the reason (s)? *(probe for minor illnesses, pain etc)*

____

____

FINANCIAL and ECONOMIC FACTORS

37. **"Are there usually additional expenses or costs such as travel, lost income, or food?"**

2 Usually	*1 Sometimes*	*0 Never*

Tick one only

Narrative____

____

37.1 **"Please estimate the total additional expenses per month"**

37.2 **"Can you usually afford to pay for them?**

2 Usually	*1 Sometimes*	*0 Never*

Tick one only

Narrative: ____

____

____

37.3 **"Have you ever had to give up anything to obtain your treatment?"**

3 Yes	*2 Possibly*	*1 Uncertain*	*0 No*

Tick one only

Narrative: ____

____

____

INFLUENCE OF HEALTHCARE PROVIDER

"Before we complete the interview, we have just a few more questions about your caregivers, your family and others."

38. **"Who are the main persons providing care for you at this clinic or group?"**

Tick all that apply

1 Doctor	
2 Nurse	
3 Counsellors/Community Healthworker	
4 Other (specify):	

39. **"Did your caregiver(s) explain your health condition to you in a way that you can understand?"**

3 Yes	2 Possibly	1 Uncertain	0 No

Tick one only

Narrative: ____

____

____

40. **"Did your cargiver(s) explain the correct use of the treatment and its effects in a way that you can understand?"**

3 Yes	2 Possibly	1 Uncertain	0 No

Tick one only

Narrative: ____

____

____

41. **"Does your caregiver(s) actively encourage you to take your treatment regularly?"**

3 Yes	2 Possibly	1 Uncertain	0 No

Tick one only

Narrative: ____

____

____

INTEPERSONAL SUPPORT and OBSTACLES TO TREATMENT

42. **"Does anyone at home usually help or encourage you to take your treatment regularly?"**

2 Usually	1 Sometimes	0 Never

Tick one only

Narrative: ____

____

____

43. **"Is there anyone at home or circumstances that usually make it difficult for you to take your treatment?**

2 Usually	1 Sometimes	0 Never

Tick one only

43.1 ***"*Please describe the circumstances and how you deal with them?"**

____

____

____

43.2. **"Is there anyone else, apart from family members, who usually help or encourage you with your treatment?"**

2 Usually	1 Sometimes	0 Never

Tick one only

Narrative: ____

____

____

43.3 **"Have you ever felt the need to hide from family members or others that you are taking treatment?"**

3 Yes	2 Possibly	1 Uncertain	0 Never

Tick one only

Narrative: ____

____

44. **“Does anyone at home or in the community usually help you treatment if yours run out?”**

2 *Usually*	1 *Sometimes*	0 *Never*

Tick one only

Narrative: ____

____

____

OTHER INFLUENCES ON TREATMENT

45. **“What do you think are the most significant problems for people with [condition] that make it difficult for them to get or take the treatment they need?”**

Narrative: ____

____

____

46. **“What suggestions do you have to help other people with [condition] get and take the treatment or important help?”**

Narrative: ____

____

____

“Thank you very much for your time and willingness to assist with this interview.”

Interview Finish Time [ ]

Inteviewer and note-taker should print names and sign and and date below. If only one person conducted interview, fill in information for interview and write ‘NA’ (not applicable) above note-taker.

______________________________ ______________________________

Interviewer **Signature and Date (**dd/mm/yyyy)

______________________________ ______________________________

Note-taker **Signature and Date (**dd/mm/yyyy)
