# Supplementary material for: Reasons for missed appointments linked to a public-sector intervention targeting patients with stable chronic conditions in South Africa: results from in-depth interviews and a retrospective review of medical records
Source: BMC Fam Pract. 2017 Aug 24;18:82. doi: 10.1186/s12875-017-0655-8 (PMC5571491; doi:10.1186/s12875-017-0655-8)
Supplement: Supplementary file 2 — Interview Schedule_KIs_GENERIC. Semi-structured interview guide for key informants. (PDF 137 kb) [file 12875_2017_655_MOESM2_ESM.pdf]

Key informant Interview Schedule – (Health facility: Pharmacist/Physician/Nurse)

Standard Information:

Name: *Position:*

Date of interview: *Consent and anonymity (preference to be documented)*

1. **Describe the following:**
   a. Your role and activities within the health facility or support group
   b. Your role in CDU implementation
2. **Which models of medicine distribution are available and/or linked to this facility?**

Model description	How many (clubs) & date started	Key person responsible	Organisation of model (e.g. frequency of meeting, location)	Other comments (Probe: advantages, challenges, patient support, opportunities for future improvement, quality of services at alternative sites)
ART clubs				
Chronic clubs				
Outreach sites				
Workplace sites				
Facility based				

3. **Describe the process of enrolling patients onto the CDU/Club programme in this health facility.**
4. **Can you relate your experiences with non-collected medicines (missed appointments) in this facility/club?**
    a. Probe: magnitude of the problem, variations by disease, season, patient characteristics (e.g. ethnic group, gender, age, social class), mode of medicines delivery, other factors.
5. **What are some of the key barriers that prohibit CDU/club patients from collecting medicines? Probe:**
    a. **Are these patients stable?**
    b. **Do patient needs change over time?**
    c. **What are the social, cultural, economic, political, environmental and other factors that might be contributing to missed appointments?**
6. **Comment on the recent move by the CDU to dispense a double supply for the festive season. Did this improve pick-up rates?** (Probe: availability of relevant statistics).
7. **Do you have site (facility or alternative site) level statistics on non-collected medicines (missed appointments)?**
    a. For which period(s)?
    b. In what format?
    c. Which variables are captured and how is this data analysed?
    d. Who is responsible for patient information management?
8. **Are there any follow-up mechanisms for tracing patients who miss appointments?** (Probe: sms reminder, home visits by Community Health Workers, collaboration with Community Based Organisations etc.)
    a. Which mechanisms are most effective? (Why?)
    b. Which mechanisms are required?
9. **In future, what mechanisms do you think should be implemented to improve collection of medicines (thereby reducing missed appointments)?**

10. **What in your view is lacking in the medicines delivery models currently employed e.g. (what should be done differently by health providers, patients, communities or other stakeholders) in order to reduce missed appointments and in the long term, to retain patients in care?**

11. **Does the CDU or your facility level programme allow for integrated care for patients with chronic co-morbidities? What are the strengths/challenges?**
12. **In your opinion, has the CDU contributed to the following:**
    a. Workload redistribution for pharmacists
    b. Overall health system efficiency, particularly at the health facility
    c. Reduced waiting times for patients
    d. Overall patient satisfaction with services/responsiveness

    (Give reasons for each response, probe for supporting data if available and for any other benefits in addition to those stated above.)

13. How does the CDU programme interact with the parallel system of medicines supply in the facility?
14. **What are some of the key lessons from CDU implementation?**
15. **What aspects of the interventions are: (a) working well? (b) not working well?**
16. **In your view, how would an ideal 'CDU' system work in order to address the prevailing challenges?**
